# Gene Alterations, Mediators, and Artificial Intelligence in Colorectal Liver Metastases

**DOI:** 10.3390/cells11142205

**Published:** 2022-07-15

**Authors:** Doris Wagner, Georgios Antonios Margonis

**Affiliations:** 1 Department of General Surgery, Medical University of Graz, 8036 Graz, Austria; 2 Department of Surgery, Memorial Sloan Kettering Cancer Center, New York, NY 10065, USA; 3 Department of General and Visceral Surgery, Charité Campus Benjamin Franklin, 10117 Berlin, Germany

In this Special Issue of *Cells*, we seek articles that focus on the study of tumor biology in order to guide the scalpel. As an example, our group published a study a few years ago that suggested that patients with *KRAS*-mutated colorectal liver metastases (CRLM) may benefit from anatomical resections [1]. These findings were later contested, and there is currently no consensus. Interestingly, new evidence has demonstrated that without the presence of co-mutated *TP53, KRAS* alone is not associated with prognosis [2,3]. Furthermore, another recent study showed that *RAS/RAF-TP53* co-alteration was the only genomic signature that predicted liver recurrence despite curative resection and local and systemic chemotherapy [3]. Thus, new studies are needed to examine whether only patients with tumors harboring certain combinations of genetic alterations benefit from anatomical resections. This highlights the importance of more meticulously assessing the extensive panel of somatic alterations [4].

To further complicate things, we now know that not all *KRAS* or *TP53* mutations are created equal. For example, missense *TP53* mutations are stratified by the evolutionary action score (EAp53) to low or high risk, and our group recently showed that the various *KRAS* point-specific mutations are associated with disparate outcomes, with median survival ranging from 30 to 80 months [5,6]. There are even point mutations associated with better survival than wild-type tumors. These findings are in concordance with research conducted by the Haigis Laboratory and others, which shows both biological and clinical differences among the many distinct *KRAS*-activating mutations [7,8,9]. Interestingly, these findings may be malignancy-agnostic as a recent study found that patients with intrahepatic cholangiocarcinoma and the G12V variant exhibit the worst outcomes [10]. This variant was also associated with the worst outcomes in a study by our group in CRLM [11]. New studies are needed to validate and further investigate these findings. For example, patients with high-risk *KRAS* mutations are less likely to undergo repeat hepatectomy for recurrence; it is unknown whether this is due to recurrence with a higher tumor burden that precludes curative intent resection or due to recurrence at an unfavorable site.

Another important question is what mediates the effects of single or combined gene alterations (and their variants) on long-term outcomes? For example, *KRAS* alterations have been associated with both a higher rate of micrometastases and a wider spread from the tumor edge [12]. In regards to combined gene alterations, a recent study by the Vauthey group from the MD Anderson Cancer Center found that *RAS/TP53* co-mutation is an independent predictor of micrometastases [13]. Of note, the study was limited by the lack of separate analyses investigating the association of *KRAS* and *TP53* alone with micrometastases. Future studies may need to be multi-institutional to allow for sufficient numbers for these stratifications. Moreover, there is no study on the association of high- vs. low-risk *KRAS* point mutations with the density and range of micrometastases.

The histopathological growth pattern of the tumor (especially the distinction between desmoplastic and non-desmoplastic subtypes) may be another mediator of the effect of gene alterations on long-term outcomes in patients with CRLM [14]. Recent studies reported that the growth pattern is an independent prognostic factor even after adjusting for *KRAS* and *BRAF* mutational status [15]. However, data on genetic alterations were available for less than half of the patients, and thus future studies with greater availability of genetic data are needed [15]. Furthermore, no studies to date have investigated the relationship between growth patterns and combinations of somatic alterations or certain high-risk *KRAS* point mutations.

Immunoregulation in the tumor microenvironment (TME) may be another mediator of the effect of gene alterations on long-term outcomes in patients with CRLM. Specifically, it was suggested that cooperative *RAS-P53* alterations may orchestrate tumor-promoting and immunosuppressive tumor–stromal–immune interactions in the TME. Others have demonstrated the positive prognostic role of tumor-infiltrating lymphocytes (TILs) and the negative prognostic role of regulatory T cells (Treg) [16,17,18].

The effect of systemic therapies on these mediators and the impact on tumor response is also largely unknown. There is a paucity of studies that specifically assess the predictive (and not the prognostic) role of the somatic alterations discussed above. One study by our group suggested that *KRAS* is prognostic only in patients who received systemic therapies, alluding to a predictive rather than prognostic role of this mutation [19]. Similarly, some studies suggested that *TP53* is not a prognostic but rather a predictive biomarker for colorectal cancer [20].

Ultimately, the implementation of precision surgery in CRLM requires that we link gene alterations with their molecular mediators. The study of somatic gene alterations in conjunction with vascular invasion, tumor growth patterns, micrometastatic disease, and host immune response is not yet possible because its importance is not well-known and only part of the liver is resected. An increased awareness and wider adoption of liver transplant (LT) may allow for the examination of the explants of patients with CRLM, as no somatic mutation with the exception of *BRAF* is a contraindication of LT. Lastly, future studies could adopt an explainable machine learning framework that harnesses more information than conventional biostatistical methods to uncover hidden, nonlinear relationships between alterations and mediators. Modern decision trees, such as OCTs, as well as methods such as LIME and SHAP, could be used to achieve this [21,22,23].
